# Secondary immunoreaction in patients with neurosyphilis and its relevance to clinical outcomes

**DOI:** 10.3389/fneur.2023.1201452

**Published:** 2023-06-06

**Authors:** Yaxiu Fang, Hong Wu, Guanghui Liu, Ziang Li, Dongmei Wang, Yuping Ning, Suyue Pan, Yafang Hu

**Affiliations:** ^1^Department of Neurology, Nanfang Hospital, Southern Medical University, Guangzhou, China; ^2^Department of Neurology, The Affiliated Brain Hospital of Guangzhou Medical University, Guangzhou, China

**Keywords:** neurosyphilis, anti-NMDAR antibody, anti-AQP4 antibody, anti-GAD65 antibody, immune impairment

## Abstract

**Background and purpose:**

Several reported cases of autoimmune conditions such as anti-NMDAR encephalitis and neuromyelitis optica (AQP4) have been considered to be potentially secondary to *Treponema pallidum* infection. Since the role of immune impairment in neurosyphilis is unclear, in this retrospective study, we examined the correlation of the immune impairment in patients with neurosyphilis with their clinical characteristics and outcomes.

**Methods:**

Clinical information was collected from patients with neurosyphilis in our center from January 2019 to December 2021. Cerebrospinal fluid (CSF) samples were subjected to indirect immunofluorescence tissue-based assay (IIF-TBA) on mouse brain sections and cell-based assay (CBA). The clinical characteristics and treatment outcomes of TBA-positive and-negative patients were compared.

**Results:**

A total number of 81 patients diagnosed with neurosyphilis were included. The results of the CBA tests showed that three cases had anti-NMDAR, AQP4, or GAD65 antibodies, respectively. By TBA test, 38 patients (38/81, 46.9%) had positive immunostains, including staining of neuronal cells in 21 cases (21/38, 55.3%), glial cells in 11 cases (11/38, 28.9%), and neuronal and glial cells in six cases (6/38, 15.8%). We then compared the clinical characteristics and treatment outcomes between the TBA-positive and-negative patients and found that TBA-positive staining was significantly correlated with syphilis antibody titers (*p* = 0.027 for serum and *p* = 0.006 for CSF) and head MRI abnormalities (*p* < 0.001 for parenchymal abnormalities and *p* = 0.013 for white matter lesions). The cognitive prognosis of TBA-positive neurosyphilis patients was significantly worse than that of TBA-negative patients (*p* < 0.001).

**Conclusion:**

The correlation between the TBA results and clinical data of our neurosyphilis patients imply the presence of secondary immune damage, which affected their prognosis. Therefore, TBA can be used as an additional biomarker for neurosyphilis patient prognosis.

## 1. Introduction

Syphilis is an infectious disease caused by *Treponema pallidum*, which can invade the nervous and immune systems and induce systemic symptoms. Currently, penicillin treatment is the main regimen for neurosyphilis therapy with doses and courses of treatment recommended by the CDC in 2001. Meanwhile, short-term oral prednisone treatment before penicillin treatment has been recommended to prevent the Jarish-Herxheimer reaction (1, 2). Nevertheless, neurosyphilis commonly occurs despite the efficiency of penicillin for syphilis treatment (3–5). The clinical outcomes of patients with *Treponema pallidum* infection of the central nervous system vary considerably among patients and syphilis stages. Antibiotic treatment can reduce syphilis titer and effectively shrink intracranial lesions in some patients, but the symptoms of a lot of patients continue to worsen during treatment, especially in the early stage of treatment (3, 4), which may be related to immune-mediated inflammation damage in the brain after neurosyphilis infection. An earlier study showed that the immune response was involved in the course of syphilis, which had no direct correlation with the local *Treponema pallidum* tissue load, but was closely related to the strength or type of the immune inflammatory response (6). A domestic investigation on neurosyphilis also established that the incidence of neurosyphilis was closely associated with immune and inflammatory responses (7). After infection with *Treponema pallidum*, strong cellular and humoral immune responses can be triggered (8). These immune responses play an important role in the elimination of *Treponema pallidum*; however, they may also lead to pathological injuries. The T cell immune response exerted by Treg and Th17 is involved in the immune damage of the central nervous system in neurosyphilis patients (7). Similarly, CXCL13/CXCR5 mediate the aggregation of peripheral blood B cells in patients with neurosyphilis, promote their humoral immune response through the formation of ectopic germinal centers, and produce antibodies targeting neuronal proteins (8–10).

Several reports of syphilis combined with autoimmune encephalitis or demyelinating diseases have been published (11–17). Notably, neurosyphilis progressed in a large number of patients even after penicillin treatment, and the disease was under control only after methylprednisolone or immunoglobulin treatment (11–14). Therefore, these findings suggest that the treatment of secondary immune damage is beneficial to the prognosis of neurosyphilis patients.

However, due to limited number of cases of autoimmune encephalitis (AE) in patients with neurosyphilis detected by cell-based assays (CBA), it is not clear whether autoimmune impairment is commonly mediated in neurosyphilis patients. In this study, we retrospectively analyzed the clinical data of 81 patients with neurosyphilis and autoantibodies in the cerebrospinal fluid (CSF) by CBA and tissue-based assay (TBA). The correlation of the immunoassay results with the clinical characteristics and treatment outcomes was also assessed.

## 2. Materials and methods

### 2.1. Patients

This study was approved by the Ethical Review Committee of the Affiliated Brain Hospital, Guangzhou Medical University (#2019-028). Signed informed consent was obtained from all patients or their family members.

Patients diagnosed with neurosyphilis in the Affiliated Brain Hospital of Guangzhou Medical University from January 2019 to December 2021 were retrospectively included. The following three inclusion criteria were applied for patients diagnosed with neurosyphilis: (1) a history of syphilis with signs and/or symptoms of nerve damage; (2) a history of syphilis with unexplained CSF abnormalities (WBC >5*10^6^/L, protein >500 mg/L); (3) a history of syphilis with positive results of a syphilis serological test, a CSF tolulized red unheated serum test (TRUST), and treponema pallidum particle agglutination (TPPA). All patients were negative for HBV, HCV, and HIV. The exclusion criteria were as follows: (1) incomplete clinical and/or relevant laboratory data; (2) severe liver or kidney disease or other infection. All patients were treated with penicillin: aqueous penicillin 18–24 million U per day, once every four hours or continuous intravenous infusion for 10–14 days, followed by intramuscular benzathine penicillin of 2.4 million U per dose once a week for three weeks. Oral prednisone (20 mg) was given once a day for 3 days before penicillin treatment.

A total number of 116 patients met the inclusion criteria. Of them, 81 patients with neurosyphilis had CSF samples and complete clinical data, including clinical examination, brain MRI, CSF analysis, and cognitive assessment (MMSE). All CSF samples were collected during the early active disease stage.

### 2.2. TBA

The use of mouse brain sections from C57/BL6J (age: 8–10 weeks) was approved by the Institutional Animal Care and Use Committee, Nanfang Hospital, Southern Medical University, Guangzhou, China. Frozen brain sections 5-μm thick were prepared as previously reported (18). Briefly, ice-cold saline-perfused mouse brain sections were fixed with isopentane-liquid nitrogen and cut to a thickness of 5 μm. For each test, 50 μL of CSF with a dilution of 1:1 was incubated with mouse brain sections at 37°C for 1 h. After washing three times with saline, each section was further incubated with 50 μL of diluted DyLight 488-labeled goat anti-human IgG secondary Ab (1:200 dilution, ab97003, Abcam, Cambridge, MA, USA) at 37°C for another 1 h. Then, the section was washed three times again and photographed under a DM3000 microscope (Leica, Wetzlar, Germany).

### 2.3. CBA

CBA is the standard method for the diagnosis of known autoantigens in patients’ samples (serum or CSF), in which HEK293 cells were used and each known autoantigen is overexpressed separately. CSF samples from patients were tested for known autoantibodies, including anti-NMDAR, LGI1, Caspr2, GABAR, AMPAR, mGluR5, GAD65, AQP4, and MOG Abs, using Euroimmun assay kits (Germany) or as previously reported (18).

### 2.4. Statistical analysis

SPSS 26.0 software was employed for statistical analysis of the data. The measurement data conforming to normal distribution were expressed as x ± s, and the two independent samples *t*-test was implemented for comparison between groups. Measurement data that did not conform to the normal distribution were represented by the mean [Interquartile range(IQR)], and the Mann–Whitney *U*-test of two independent samples was applied for comparison between groups. The statistical data were expressed as frequency and percentage, and the chi-squared test or Fisher’s exact test was performed for comparison between groups. Logistic regression and linear regression analysis were used to analyze the univariate variables with statistical differences. For all analyses, *p* < 0.05 was considered statistically significant.

## 3. Results

### 3.1. Epidemiology and clinical characteristics

**Figure 1:**
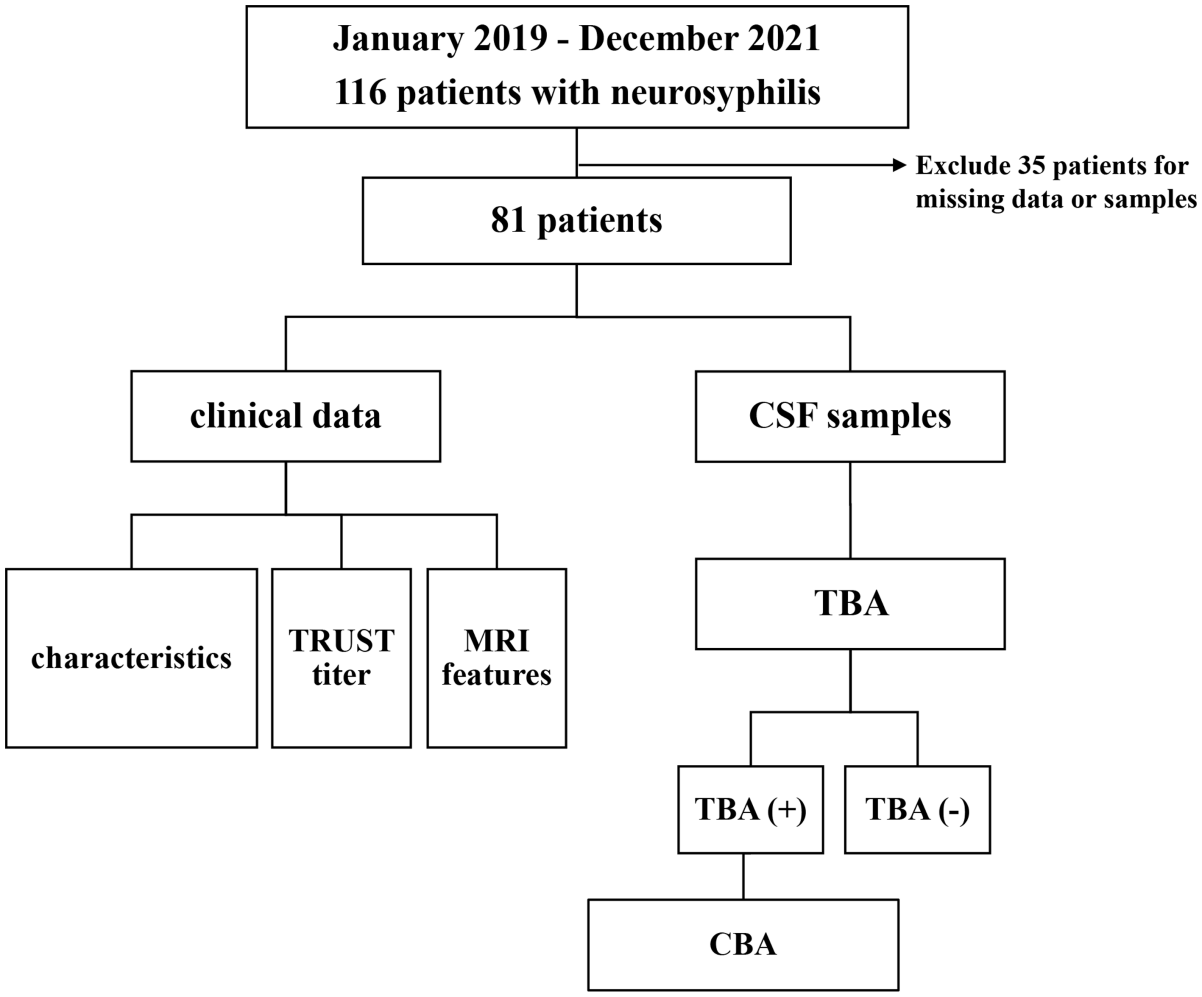
Flow chart of the study of 81 patients with neurosyphilis. CSF, cerebrospinal fluid; TRUST, tolulized red unheated serum test; MRI, magnetic resonance image; TBA, tissue-based assay; CBA, cell-based assay; NMDAR, Anti-N-methyl-D-aspartate-receptor; GAD65, glutamic acid decarboxylase 65; AQP4, aquaporin-4.

A total number of 116 cases were diagnosed with neurosyphilis from January 2019 to December 2021, of which 81 were included in this study. Clinical retrospective analysis was performed with all cases that met the inclusion criteria, and CSF samples were subjected to CBA and TBA tests. The study flowchart and results are presented in Figure 1.

The epidemiological and clinical characteristics of the 81 neurosyphilis patients who met the inclusion criteria are displayed in Table 1. Their age range is from 47.75–60 years. The main clinical symptoms were memory decline, in 78 (96.3%) cases, and mental behavior abnormalities, in 76 (93.8%) cases; cases with other symptoms were as follows: dizziness in two cases (2.5%), headache in two cases (2.5%), limb weakness in five cases (6.2%), epilepsy in four cases (4.9%), insomnia in eight cases (9.9%), decreased visual acuity in two cases (2.5%), ataxia in one case (1.2%), and paresthesia in one case (1.2%). Before treatment, the CSF had a WBC count of 8 (2.75–22) 10^6/L and total protein of 0.65 (range 0.51–1.03) g/L. MRI showed parenchymal abnormalities or inflammatory lesions in 28 cases (25.2%), brain atrophy in 56 cases (69.1%), and leukoencephalopathy in 11 cases (9.9%).

**Table 1:** Demographic and clinical data of 81 neurosyphilis patients.

Characteristic	Value
Age, year, median (IQR) Symptom duration, month, median (IQR)	54 (47.75–60) 18 (8.75–36)
Main clinical features	
Memory deterioration, *n* (%)	78 (96.3)
Mental disorder and abnormal behavior, *n* (%)	76 (93.8)
Dizziness, *n* (%)	2 (2.5)
Headache, *n* (%)	2 (2.5)
Lacking in strength, *n* (%)	5 (6.2)
Epilepsy, *n* (%)	4 (4.9)
Insomnia, *n* (%)	8 (9.9)
Visual deterioration, *n* (%)	2 (2.5)
Disorder of ataxia, *n* (%)	1 (1.2)
Paresthesia of lower limbs, *n* (%)	1 (1.2)
CSF diagnostics	
WBC, 10^6^/L, median (IQR)	8 (2.75–22)
Protein, g/L, median (IQR)	0.65 (0.51–1.03)
Syphilis titer	
Serum TRUST titer, median (IQR)	12 (4–32)
CSF TRUST titer, median (IQR)	1.5 (0–4)
MRI	
Parenchymal abnormalities, *n* (%)	28 (25.2)
Cerebral atrophy, *n* (%)	53 (65.4)
White matter lesions, *n* (%)	11 (9.9)
MMSE, median (SD)	14.06 (0.44)

CSF, cerebrospinal fluid; WBC, white blood cells; TRUST, tolulized red unheated serum test; MRI, magnetic resonance image; MMSE, Mini-mental State Examination.

### 3.2. Immunostaining of mouse brain sections in CSF by TBA

**Figure 2:**
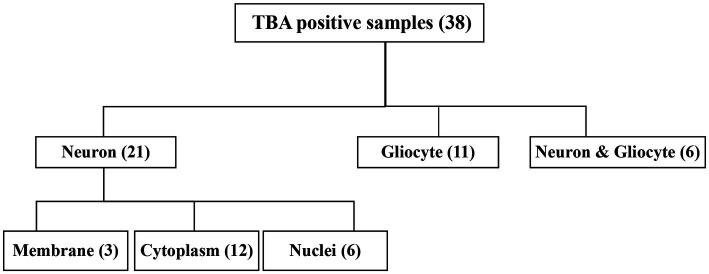
Flow chart of the TBA. TBA, tissue-based assay.

**Figure 3:**
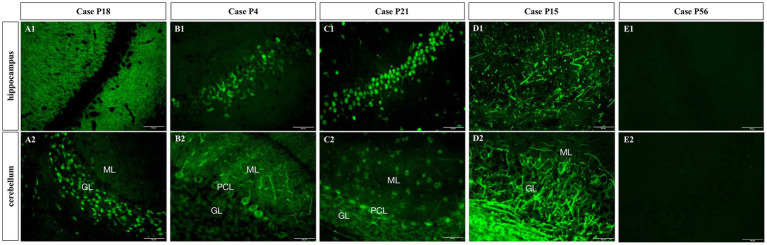
Subcellular localization of TBA-positive staining. **(A1,A2)** Cell surface of neurons; **(B1,B2)** Cytoplasm of neurons; **(C1,C2)** Nuclei; **(D1,D2)** Gliocytes; **(E1,E2)** TBA-negative case example. GL, granular layer; ML, molecular layer; PCL, Purkinje cell layer. Scale bars represent 50 μm.

The CSF samples of 81 patients were tested with TBA, as can be seen in the flowchart in Figure 2. There were 38 samples (38/81, 46.9%) with positive immunostaining to the mouse brain cells. According to the cellular morphology of the staining, the positive staining was classified as neuronal cells in 21 cases (including cell surface in three cases, nuclei in six cases, and cytosol in 12 cases), glial cells in eleven cases (11/38, 28.9%), and both neuronal cells and gliocytes in six cases, as illustrated in Figure 3.

### 3.3. Three neurosyphilis patients with known autoimmune encephalitis antibodies

**Figure 4:**
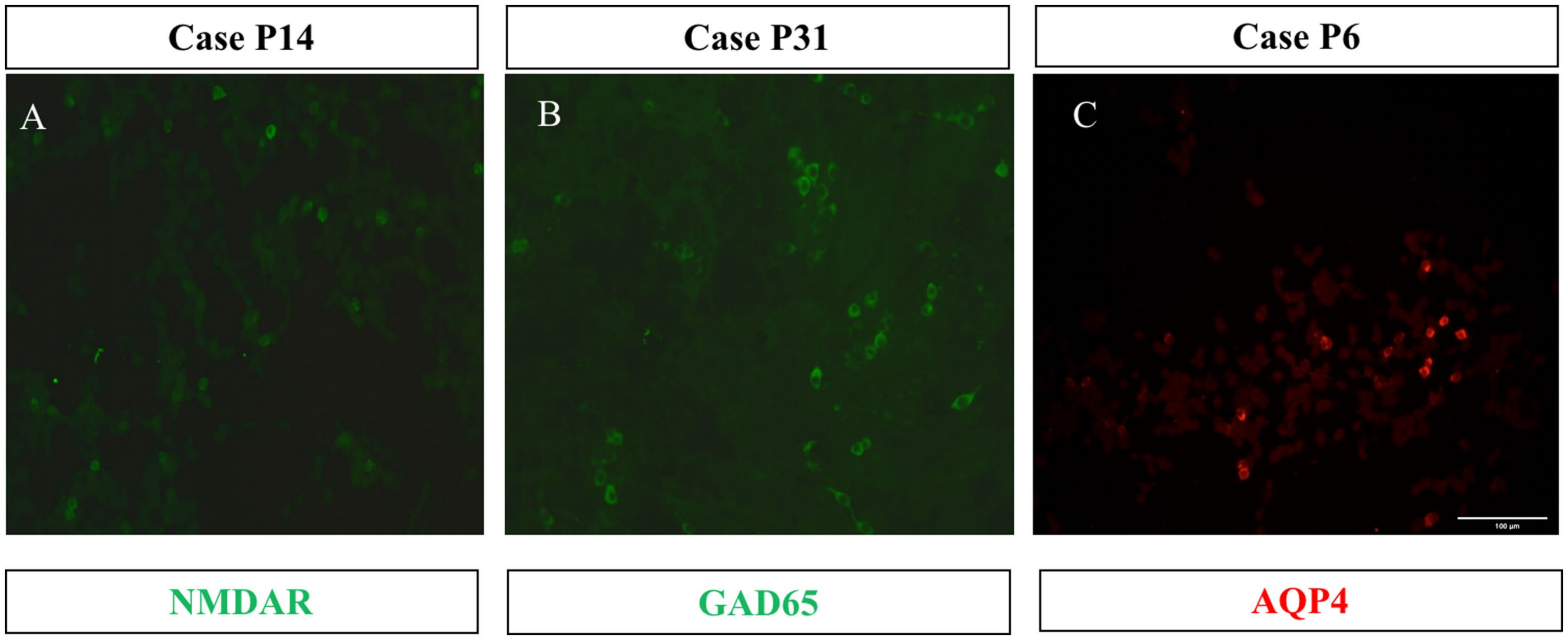
Three cases with CBA-positive results. HEK293 cells expressed the full length of the cDNA fragment of NMDAR **(A)**, GAD65 **(B)**, and AQP4 **(C)**. 1:1 dilution of CSF specimens from Patients 14, 31, and 6 were tested. The second antibody was DyLight 488-labeled (green) or DyLight 550-labeled (red) goat anti-human IgG. Scale bars represent 100 μm.

The known membrane antibodies (anti-NMDAR, LGI1, Caspr2, GABABR, AMPA_A/B_R, and mGluR5) and intracellular antibody(GAD65)were analyzed in 21 patients with TBA staining of neuronal cells. Among of them, one case (P14) was positive for the anti-NMDAR antibody and another case (P31) was positive for the anti-GAD65 antibody. These two patients also had serum and CSF tests when they were hospitalized: P14 had a CSF titer of 1:1 anti-NMDAR antibody (Figure 4A) and a serum titer of 1:10; P31 had a CSF titer of 1:1 anti-GAD65 antibody (Figure 4B) and a serum titer of 1:10. Anti-MOG and AQP4 Abs were analyzed in the CSF samples of 11 patients with TBA staining in their glial cells; CBA identified only one case (P6) positive for the anti-AQP4 antibody with a titer of 1:1 (Figure 4C). Serum samples for case P6 are unavailable. Six cases had both neuronal and astrocyte patterns, and no known antibodies were detected.

**Figure 5:**
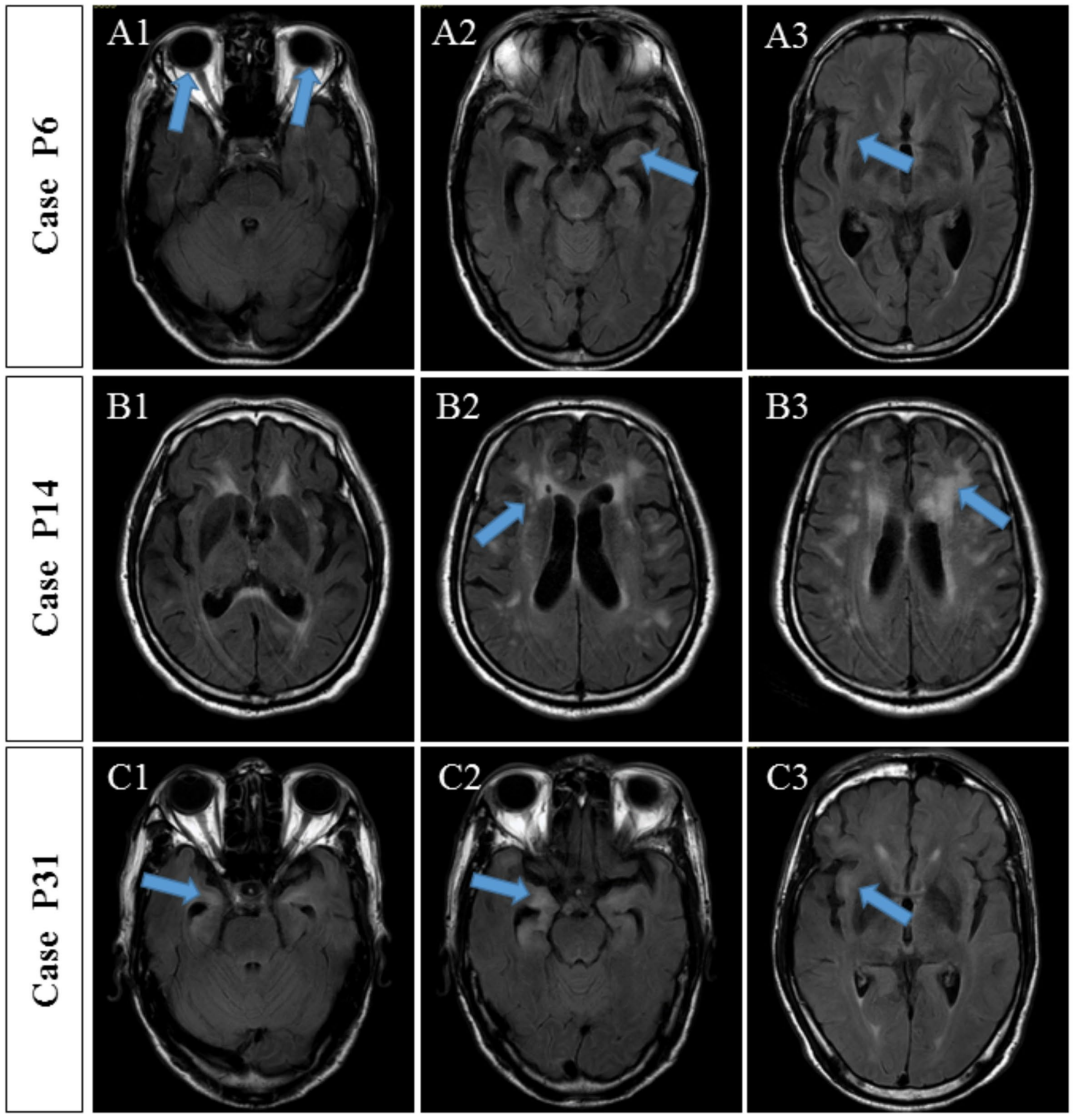
MRI findings of three patients with autoantibody-positive neurosyphilis. **(A)** Case P6, MRI showed multiple abnormal signal shadows in the bilateral temporal cortex and subcortex. **(B)** Case P14, MRI showed multiple ischemic lesions in the frontotemporal parietal lobe and the lateral ventricle and brain atrophy. **(C)** Case P31, MRI showed multiple abnormal signals in the bilateral frontal, temporal, and insular lobes, indicating inflammatory lesions and brain atrophy.

The detailed clinical manifestations of the three patients are described as follows. P6 (positive anti-AQP4 antibody) was admitted to hospital due to excessive excited talking and decreased sleep for 10 days. The serum and CSF TRUST titers were 1:32 and 1:8, respectively. CSF analysis showed a WBC of 50 × 10^6^/L and a protein level of 0.67 g/L. Brain MRI revealed multiple abnormal signal shadows in the bilateral temporal cortex and subcortex (Figure 5A), MMSE increased from 12 to 18 points post-penicillin treatment. No visual or spinal symptoms were found. The significance of AQP4 Ab positivity was unclear and accompanied AQP4-associated disorder was uncertain.

P14 with the anti-NMDAR antibody was hospitalized for a 10-month memory decline, 5-month lower limb weakness, and 1-month abnormal mental behavior. Upon admission, the serum and CSF TRUST titers were 1:128 and 1:4, respectively. CSF analysis showed increased WBC (3 × 10^6/L) and protein level (0.66 g/L). Brain MRI showed multiple ischemic lesions in the frontotemporal parietal lobe and the lateral ventricle and brain atrophy (Figure 5B). The MMSE score was 7 points. The symptoms continued to progress after the penicillin treatment. Although accompanied anti-NMDAR encephalitis were diagnosed, the patient’s condition meant he was unable to accept glucocorticoid treatment and he refused to receive immunoglobulin treatment due to economic situation. Three weeks later, the patient complicated with severe pneumonia, respiratory failure, and acute coronary syndrome and was transferred to another hospital by his family.

P31 (positive anti-GAD65 antibody) was hospitalized due to mental disorder and abnormal behavior accompanied by memory decline for 3 years and paroxysmal convulsions for 10 days. Serum and CSF TRUST titers were 1:16 and 1:4, respectively. Pleocytosis (WBC of 56 × 10^6/L) and an increased protein concentration (1.64 g/L) were found in the CSF. Brain MRI showed multiple abnormal signals in the bilateral frontal, temporal, and insular lobes, indicating inflammatory lesions and brain atrophy (Figure 5C). There was no obvious improvement in the brain MRI-detected abnormalities after the penicillin treatment. Nevertheless, the cognitive impairment was improved and the MMSE score increased from 12 to 20 points.

### 3.4. Correlation analysis between TBA-positive and clinical features of neurosyphilis patients

**Figure 6:**
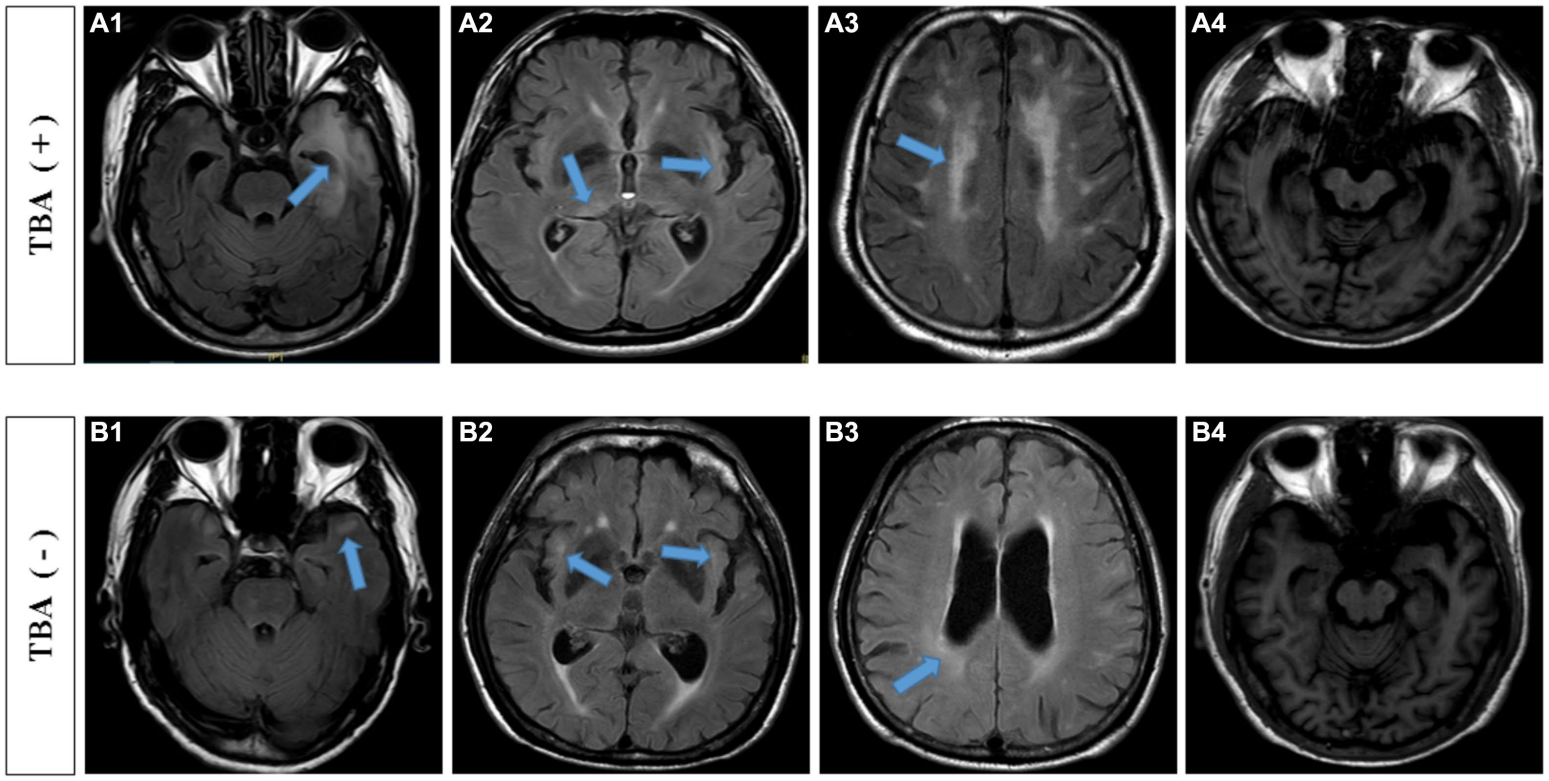
MRI findings of TBA-positive and-negative neurosyphilis patients. TBA-positive neurosyphilis patients: **(A1,A2)** parenchymal abnormalities in the frontotemporal and hippocampal regions on the cranial MRI images. **(A3)** white matter lesions, **(A4)** diffuse cerebral atrophy and hippocampal atrophy. TBA-negative neurosyphilis patients: **(B1,B2)** parenchymal abnormalities in the frontotemporal and hippocampal regions on the cranial MRI images. **(B3)** white matter lesions, **(B4)** diffuse cerebral atrophy and hippocampal atrophy. MRI, magnetic resonance image; TBA, tissue-based assay.

The most common symptoms of the 38 TBA-positive patients were cognitive impairment (35 cases, 92.1%) and abnormal mental behavior (37 cases, 97.4%). Other symptoms included dizziness (one case), limb weakness (two cases), convulsions (three cases), insomnia (eight cases), vision loss (one case), ataxia (one case), and paresthesia of the lower extremities (one case). As shown in Table 2, the duration in the TBA-positive group was significantly shorter than that in the TBA-negative group, *p* = 0.007. There were no significant differences in the pleocytosis and protein levels of CSFs between the TBA-positive and TBA-negative groups. However, both the serum and CSF syphilis titers in the TBA-positive group were higher than those in the TBA-negative group, with significant differences between the groups of *p* = 0.027 and 0.006, respectively. MRI parenchymal abnormalities in the frontotemporal and hippocampal regions in the TBA-positive group were significantly higher (57.9%) than those in the TBA-negative group (14%) (*p* < 0.001, Table 2; Figure 6). A significant difference was found in the white matter lesions (*p* = 0.013) but not diffuse cerebral atrophy between the two groups. The MMSE scores of the patients in the TBA-positive group was lower than that in the TBA-negative group, with a significant difference (*p* < 0.001) between them (Table 2).

**Table 2:** Comparison of clinical features between positive and negative TBA groups.

Characteristic	TBA(+) (*n* = 38)	TBA(−) (*n* = 43)	*p* value
Age, year, median (IQR)	55 (48–60)	54 (47–59.5)	0.943
Duration, months, median (IQR)	12 (6–31.5)	24 (11.25–58.5)	0.007
CSF			
WBC, 10^6^/L, median (IQR)	11 (3–38)	8 (2–13.5)	0.895
Protein, g/L, median (IQR)	0.72 (0.60–0.98)	0.58 (0.47–0.70)	0.489
Syphilis titer			
Serum TRUST titer, median (IQR)	24 (8–32)	4 (2–16)	0.027
CSF TRUST titer, median (IQR)	4 (1–5)	0 (0–2)	0.006
MRI			
Parenchymal abnormalities, *n* (%)	22 (57.9)	6 (14)	<0.001
Cerebral atrophy, *n* (%)	25 (65.8)	28 (65.1)	0.949
White matter lesions, *n* (%)	9 (23.7)	2 (4.7)	0.013
MMSE, median (SD)	12.816 (0.750)	15.25 (0.390)	<0.001

TBA, tissue-based assay; CSF, cerebrospinal fluid; WBC, white blood cells; MMSE, Mini-mental State Examination; MRI, magnetic resonance image; TRUST, tolulized red unheated serum test.

Multivariate logistic regression analysis of factors affecting severe cognitive impairment (MMSE≤10) (Table 3) revealed that a TBA-positive result was an independent risk factor (*p* = 0.011) for severe cognitive impairment, whereas a positive syphilis TRUST result was not. These results suggest that TBA positivity plays a greater role in the occurrence of severe cognitive impairment in patients with neurosyphilis than that of a positive TRUST result.

**Table 3:** Multivariate logistic regression analysis of factors influencing severe cognitive impairment (MMSE ≤ 10).

Characteristic	OR (95% CI) (univariate)	*p* value	OR (95% CI) (multivariate)	*p* value
Serum TRUST titer	1.023 (1.004–1.042)	0.016	1.014 (0.994–1.036)	0.146
TBA	10.66 (2.219–51.217)	0.003	8.181 (1.632–41.017)	0.011

MMSE, Mini-mental State Examination; OR, odds ratio; CI, confidence interval; TRUST, tolulized red unheated serum test; TBA, tissue-based assay.

In addition, multivariate regression analysis of the parenchymal lesions revealed that a TBA-positive result, but not the serum syphilis titer, was independent risk factor as shown in Tables 2, 4. However, immunostaining in different cell types, neuronal cells or glial cells, had no significant correlation with the site of parenchymal lesions in the brain MRI (Table 5).

**Table 4:** Multivariate logistic regression analysis of factors influencing MRI parenchymal abnormalities.

Characteristic	OR (95% CI) (univariate)	*p* value	OR (95% CI) (multivariate)	*p* value
Age	0.942 (0.897–0.989)	0.017	0.931 (0.873–0.994)	0.031
Serum TRUST titer	1.021 (1.003–1.040)	0.025	1.006 (0.984–1.029)	0.590
TBA	8.479 (2.890–24.880)	<0.001	7.847 (2.227–27.644)	0.001

MRI, magnetic resonance image; OR, odds ratio; CI, confidence interval; TRUST, tolulized red unheated serum test; TBA, tissue-based assay.

**Table 5:** Correlation between different TBA colored cell types and MRI lesion sites.

Characteristic	Gliocyte (*n* = 16)	Neuron (*n* = 27)	*p* value
Parenchymal abnormalities, *n* (%)	10 (62.50)	14 (51.85)	0.417
Cerebral atrophy, *n* (%)	12 (75.00)	18 (66.67)	0.262
White matter lesions, *n* (%)	4 (25.00)	6 (22.22)	0.938

MRI, magnetic resonance image; TBA, tissue-based assay.

### 3.5. Correlation analysis between treatment outcome and positive TBA results

In terms of treatment, all 81 patients received penicillin shock therapy. Although prednisolone was administered for three days prior to penicillin use, none received intravenous methylprednisone and/or intravenous immunoglobulin. The pre-disease state of cognition was basically restored in three of the TBA-positive patients, whereas the other 35 patients had only partial cognitive improvement. However, 28 of the TBA-negative patients returned to normal cognition, and 15 patients showed partial improvement. The cognitive outcomes of TBA-negative neurosyphilis patients were better than those of the TBA-positive neurosyphilis patients, with a significant correlation between the two groups (Table 6).

**Table 6:** Association between positive TBA and the treatment effect of neurosyphilis.

Variable	Total	TBA (+)	TBA (−)	*p* value
	*N* = 81	*N* = 38	*N* = 43	
Age(year)	54 (47.75–60)	55 (48–60)	54 (47–59.5)	0.943
Gender(male, *n*%)	62 (76.5)	33 (86.8)	29 (67.4)	0.004
Duration(months)	18 (8.75–36)	12 (6–31.5)	24 (11.25–58.5)	0.007
MMSE(Before treatment)	14.06 (0.44)	12.8 (0.750)	15.25 (0.39)	<0.001
MMSE(After treatment)	17.83 (0.54)	16.3 (0.971)	19.20 (0.33)	<0.001

TBA, tissue-based assay; MMSE, Mini-mental State Examination.

## 4. Discussion

To explore the secondary immune damage of the syphilis patients in this study, we analyzed the clinical data of 81 patients with definite diagnosis of neurosyphilis. Additionally, we performed TBA and CBA analyses of the CSF specimens from the patients and analyzed the correlation in their clinical characteristics and laboratory results. We found that 38 out of 81 patients (46.9%, 38/81) had TBA-positive results. Further, CBA testing identified known autoantibodies in three cases, the anti-NMDAR, anti-AQP4, and anti-GAD65 antibodies. To our knowledge, a positive anti-GAD65 antibody was detected for the first time in a patient with neurosyphilis. Importantly, we found that a positive TBA was associated with the syphilis titer, abnormal changes in the brain MRI, and prognosis of cognitive impairment.

In this study, three patients tested positive for known antibodies. Patient 6 with anti-AQP4 antibody showed no symptoms of encephalomyelitis or optic neuritis except typical clinical symptoms of syphilis and MRI imaging findings; no serum tests were conducted. The treatment administered was effective. Therefore, the significance of its complication with AQP4 is unclear and the case will be followed up. The clinical manifestations of Patient 14 with NMDAR encephalitis overlapped, and MRI imaging showed the characteristics of limbic encephalitis. The symptom progress continued after the penicillin treatment. Together with the positive anti-NMDAR antibody in CSF and serum, this patient was diagnosed with secondary NMDAR encephalitis to neurosyphilis. Unfortunately, the patient was unable to receive immunotherapy in our hospital. The anti-GAD65 antibody was found in the serum and CSF of Patient 31. The clinical manifestations overlapped with anti-GAD-65 encephalitis. Multiple abnormal signals in the frontal, temporal, and insula lobes on the bilateral brain MRI were considered to be inflammatory lesions. Although the brain MRI changes were not obvious after the penicillin treatment, the cognitive impairment was improved, and the MMSE score increased from 12 points to 20 points. Due to most cases of anti-GAD65 encephalitis having high titer antibodies and this case having low titer anti-GAD65, we did not thus diagnose Patient 31 with anti-GAD65 encephalitis and put the patient on the follow-up list. In recent years, cases of neurosyphilis complicated with autoimmune encephalitis have been reported (11–17). For example, Qin et al. (13) reported a case of neurosyphilis combined with anti-NMDAR antibodies, and immunotherapy with methylprednisolone and immunoglobulin improved the patient’s condition. In a case of neurosyphilis complicated with AQP4-associated optic neuromyelitis, disease progression was controlled only after treatment with penicillin combined with the administration of immunosuppressive agents (14). Thus, once AE secondary to neurosyphilis is diagnosed, immunotherapy may be of benefit to the patient.

The significance of this study lies in the established significant correlation between positive TBA results and abnormal brain MRI or worse prognosis of cognitive impairment. In the TBA-positive neurosyphilis patients, 57.9% of patients had parenchymal abnormalities in the frontotemporal and hippocampus region, 55.3% had cerebral infarction/ischemia, and 42.1% had white matter lesions. The MRI abnormalities showed significant differences between the TBA-positive group and the TBA-negative group, suggesting that the TBA test is an indicator that can facilitate the judgment of the severity of the nervous system damage. In addition, among of the 38 TBA-positive patients, only three patients had a return to basic cognitive levels whereas the other 35 had only a partial improvement of their cognitive impairment. In the TBA-negative group, 28 patients had cognitive normalcy, and 15 had partial improvement. The prognosis of cognitive impairment in the TBA-negative neurosyphilis patients was better than that in the TBA-positive patients, with a significant correlation between the two groups. Therefore, these findings suggest that secondary immune damage occurs in neurosyphilis patients and TBA can serve as an auxiliary indicator to judge the prognosis. A TBA-positive reaction may imply the presence of immune-mediated inflammatory lesions or unknown autoantibodies. A limitation of this study is the small size of patients and short time of follow-up. Therefore, in the treatment of TBA-positive neurosyphilis patients, whether it is necessary to add immunotherapy for better prognosis is worth investigating with larger samples.

The mechanism by which syphilis leads to secondary immune impairment is unknown. Various hypotheses have been proposed, including the exposure of autoantigens to damaged tissue caused by pathogen infection (19, 20), autoimmune cross-reaction of pathogen cell receptors (21), activation of autoimmune response cells by pathogens (22), and change to the immune state of the host or the regulatory point of immune tolerance (23–32). In addition, the destruction of the blood–brain barrier can also facilitate the invasion of *Treponema pallidum* into the central nervous system, resulting in an *in situ* immune response, and ultimately the synthesis of immunoglobulin in the sheath (33). In conclusion, the immune response of the pathogenic autoantibodies or T cells might be part of the pathogenesis of secondary nervous system damage in neurosyphilis. Nevertheless, more comprehensive and larger-sample studies are needed to clarify the significance of secondary immune damage.

## 5. Conclusion

Patients with neurosyphilis may have secondary immune damage. The CBA test may identify the known pathogenic autoantibodies and the TBA method provides information on possible immune-mediated impairment. In addition, the TBA result is an indicator of the severity of the disease and outcome prediction.

## Data availability statement

The original contributions presented in the study are included in the article/supplementary material, further inquiries can be directed to the corresponding authors.

## Ethics statement

The studies involving human participants were reviewed and approved by the Ethical Review Committee of the Affiliated Brain Hospital, Guangzhou Medical University. The patients/participants provided their written informed consent to participate in this study. The animal study was reviewed and approved by the Institutional Animal Care and Use Committee, Nanfang Hospital, Southern Medical University, Guangzhou, China.

## Author contributions

YH, SP, YN, and YF designed the study and drafted the manuscript. YF took care of the index patient and was responsible for the clinical data preparation. HW and GL conducted the experiments and took the TBA images. All authors contributed to the article and approved the submitted version.

## Funding

This work was supported by the National Natural Science Foundation of China (82201505) and the Clinical Research Project of Nanfang Hospital (2021CR020).

## Conflict of interest

The authors declare that the research was conducted in the absence of any commercial or financial relationships that could be construed as a potential conflict of interest.

## Publisher’s note

All claims expressed in this article are solely those of the authors and do not necessarily represent those of their affiliated organizations, or those of the publisher, the editors and the reviewers. Any product that may be evaluated in this article, or claim that may be made by its manufacturer, is not guaranteed or endorsed by the publisher.

## Abbreviations

AE: autoimmune encephalitis
AMPAR: A-amino-3-hydroxy-5-methyl4-isoxazolepropionicacid receptor
AQP4: aquaporin-4
Caspr2: contactin-associated protein
CBA: cell-based assay
CDC: Centers for Disease Control
CSF: cerebrospinal fluid
CXCL13/CXCR5: chemokine CXC ligand 13/chemokine CXC receptor 5
GABAR: gammaaminobutyric acid receptor
GAD65: glutamic acid decarboxylase 65
HBV: hepatitis B virus
HCV: hepatitis C virus
HIV: human immunodeficiency virus
LGI1: leucine-rich glioma inactivated 1
mGluR5: metabotropic glutamate receptor 5
MRI: magnetic resonance image
MOG: myelin oligodendrocyte glycoprotein
MMSE: mini-mental state examination
NMDAR: N-methyl-D-aspartate-receptor
TBA: tissue-based assay
Treg: regulatory T cells
Th17: T helper cell 17
WBC: white blood cells
TRUST: tolulized red unheated serum test
TPPA: treponema pallidum particle agglutination
